# A Pragmatic Assessment of Google Translate for Emergency Department Instructions

**DOI:** 10.1007/s11606-021-06666-z

**Published:** 2021-03-05

**Authors:** Breena R. Taira, Vanessa Kreger, Aristides Orue, Lisa C. Diamond

**Affiliations:** 1 Olive View-UCLA Medical Center, Sylmar, CA USA; 2 Memorial Sloan Kettering Cancer Center, New York, NY USA

**Keywords:** communication barriers, translation, machine translation, language services

## Abstract

**Background:**

Because many hospitals have no mechanism for written translation, ED providers resort to the use of automated translation software, such as Google Translate (GT) for patient instructions. A recent study of discharge instructions in Spanish and Chinese suggested that accuracy rates of Google Translate (GT) were high.

**Study Objective:**

To perform a pragmatic assessment of GT for the written translation of commonly used ED discharge instructions in seven commonly spoken languages.

**Methods:**

A prospective assessment of the accuracy of GT for 20 commonly used ED discharge instruction phrases, as evaluated by a convenience sample of native speakers of seven commonly spoken languages (Spanish, Chinese, Vietnamese, Tagalog, Korean, Armenian, and Farsi). Translations were evaluated using a previously validated matrix for scoring machine translation, containing 5-point Likert scales for fluency, adequacy, meaning, and severity, in addition to a dichotomous assessment of retention of the overall meaning.

**Results:**

Twenty volunteers evaluated 400 google translated discharge statements. Volunteers were 50% female and spoke Spanish (5), Armenian (2), Chinese (3), Tagalog (4), Korean (2), and Farsi (2). The overall meaning was retained for 82.5% (330/400) of the translations. Spanish had the highest accuracy rate (94%), followed by Tagalog (90%), Korean (82.5%), Chinese (81.7%), Farsi (67.5%), and Armenian (55%). Mean Likert scores (on a 5-point scale) were high for fluency (4.2), adequacy (4.4), meaning (4.3), and severity (4.3) but also varied.

**Conclusion:**

GT for discharge instructions in the ED is inconsistent between languages and should not be relied on for patient instructions.

## INTRODUCTION

Patients with limited English proficiency (LEP) have low rates of understanding of appointment type and medications^1^, higher rates of medication errors^2^, and unplanned return visits to an emergency department.^3^ The discharge process is a particularly important point in terms of patient–provider communication. Written discharge instructions contain critical information about the patient’s diagnosis, treatment plan, and follow-up.

Whereas most hospitals in the USA have access to spoken language assistance via phone interpreters, a gap exists in the capacity for written translation.^4^ Many electronic health records (EHRs) have pre-written patient education sheets for specific diagnoses such as “Upper Respiratory Infection” in a variety of languages and providers can easily use these to provide written materials in the patient’s preferred language. The challenge, however, is when the provider must convey patient specific instructions such as “Come to the ophthalmology clinic at 8 am on Thursday and bring your records from the outside hospital.” Frequently, there is no mechanism for requesting written translations in the acute setting. While the optimal response in this situation is to write the patient’s discharge instructions in English and have the instructions verbally interpreted to the patient using a certified health care interpreter, many providers resort to the use of machine translation for efficiency. Google Translate is an increasingly popular option for written translation^5,6^ and, in some hospital systems, has become the go-to source of written translations, especially for patient-specific discharge instructions given to LEP patients. In a frequently cited study, Patil and Davies found that Google Translate was only 57% accurate and concluded that it could not be trusted for the translation of medical phrases.^7^ This 2014 study, however, was completed prior to an improvement in the Google Translate algorithm^8^ and the phrases chosen for evaluation were in British English rather than the English used in the USA and thus may represent a mis-estimation of the accuracy of Google Translate for discharge instructions in US hospitals. Conversely, in an abstract published in 2010, Khanna et al. found that Google Translate was relatively accurate for patient education, but they assessed only Spanish.^9^ Recently, Khoong et al. studied the use of Google Translate (GT) for ED discharge instructions in Spanish and Chinese and concluded that GT had high accuracy and GT translations can supplement but not replace written English instructions and should include a warning about potentially inaccurate instructions.^10^ This study, however, only assessed Spanish and Chinese—two of the most common languages spoken—and used professional translators to evaluate the translations. Because Google Translate improves its algorithms from user feedback, it would be expected to perform differently for more common languages compared to languages with fewer speakers. In addition, the understanding of a professional interpreter who is trained in the nuances of both languages may not be representative of the understanding of the average community member who presents to the ED for care.

The primary objective of this study was to perform a pragmatic assessment of the accuracy of GT for the written translation of commonly used ED discharge instructions given to patients in each of the most common languages spoken by LEP patients as assessed by bilingual community members. The secondary objective is to compare the performance of GT between languages.

## METHODS

### Study Design

We selected frequently used instructions written when discharging a patient from an emergency department visit that convey critical information about the treatment or follow-up plan. We constructed a list of candidate statements that reflect statements most often used in free-form written patient instructions in our ED. The candidate statements were then reviewed by a group of practicing ED clinicians (MD, NP, and RN) not involved in the study. The group was asked to comment and, based on the responses, a final group of 20 ED discharge instructions were chosen. The five most frequently spoken languages in Los Angeles County were extracted for the study (Spanish, Chinese (including Mandarin and Cantonese), Tagalog (including Filipino), Vietnamese, and Korean). Armenian and Farsi are very common in our ED and were added not only because of the direct utility of the data in our setting, but also to compare the accuracy of GT for these languages of lesser diffusion. Each of the 20 discharge instruction statements was then translated using GT into all 7 of the target languages.

### Subjects

Volunteer native speakers of each of the target languages were identified. Volunteers were included if they were native speakers of one of the target languages (not heritage speakers), currently fluent in English, and could read both languages. Participants were excluded if they worked in any aspect of health care or were a professional interpreter or linguist to assure a pragmatic assessment of these instructions in community members. IRB approval was obtained before the initiation of the research.

### Measures

Basic demographics of the participants included gender, years in the USA, self-reported ability to understand English and self-reported ability to understand the target language. In addition, we asked each volunteer to complete a 4-question acculturation scale.^11^ Although this scale has been validated for study participants of Hispanic origin, it has similar properties to that of validated tools for other groups.^12^

### Outcomes

Participants received a worksheet with each of the Google Translated instruction statements in their native language and were asked to verbally explain to the research team member the meaning of each of the statements in English. The primary outcomes were whether the intent of the statement was retained (yes/no). The bilingual volunteer then used the machine translation scoring rubric to evaluate each statement. Volunteers were given standardized instructions and oriented to the rubric. This rubric contains a 5-point Likert scale for fluency, adequacy, meaning, and severity and is standard for rating machine translation.^13^ The volunteers gave their rating on fluency, adequacy, and meaning and the research team member (an MD or NP) chose the clinical severity based on the explanation given by the volunteer.

### Analysis

Descriptive statistics (proportions with 95% confidence intervals) were used for the accuracy rate of Google Translate overall for simple discharge instructions (statements in which the meaning was retained/total statements) and for each language. Scores for each of the rubric categories were reported using means.

## RESULTS

Between March 5, 2019, and Feb 6, 2020, we recruited a total of twenty participants who evaluated twenty discharge instructions each for a total of 400 discharge instructions examined. There were an equal number of male and female volunteers. They spoke Spanish (5), Armenian (2), Chinese (3), Tagalog (4), Vietnamese (2), Korean (2), and Farsi (2). Their mean years living in the USA was 23 (range 3–47). All self-reported that they spoke English well (4/20) or very well (16/20) and the target language very well (18/20) or well (2/20). Rates of acculturation were high (see Table 1). Mean scores for fluency adequacy, meaning, and severity were high, ranging from 4.2 to 4.4 on a 5-point Likert scale but varied by language (see Table 2). Overall, GT accurately conveyed the meaning of 330/400 (82.5%) instructions examined but the accuracy varied by language from 55 to 94%. Some of the translation errors reported by the volunteers made the GT translations non-sensical (see Table 3 for illustrative examples).

**Table 1:** Participant Demographics

Native language	Spanish	5 (25%)
	Armenian	2(10%)
	Chinese	3 (15%)
	Tagalog	4 (20%)
	Vietnamese	2 (10%)
	Korean	2 (10%)
	Farsi	2 (10%)
Female		10/20 (50%)
Mean years in the USA		23.7 years (range 3-47)
English proficiency	Very well	16/20 (80%)
	Well	4/20 (20%)
Target language proficiency	Very well	18/20 (90%)
	Well	2/20 (10%)
Acculturation scale		
Read and speak	English better than native	3 (15%)
	Both equally	14 (70%)
	Native better than English	3 (15%)
Speak at home	More English than native language	3 (15%)
	Both equally	9 (45%)
	More native language than English	3 (15%)
	Only native language	5 (25%)
Think	More English than native	7 (35%)
	Both equally	7 (35%)
	More native than English	3 (15%)
	Only target	3 (15%)
Speak with friends	Only English	2 (10%)
	More English than native	2 (10%)
	Both equally	13 (65%)
	More native than English	3 (15%)

**Table 2:** Mean Fluency, Adequacy, Meaning, Severity on 5-Point Likert Scales, and Overall Accuracy (# Accurate Statement/# Statements Evaluated) by Language

Language (# participants)	Fluency	Adequacy	Meaning	Severity	# Accurate statements/#statements evaluated	*Accuracy rate*	95%CIs
Spanish (5)	4.8	4.8	4.8	4.8	94/100	94%	87.4–97.7
Armenian (2)	3.7	3.6	3.3	3.4	22/40	55%	38.4–70.7
Chinese (3)	4.1	4.5	4.1	4.1	49/60	81.7%	69.6–90.5
Tagalog (4)	4.4	4.7	4.6	4.7	54/60	90%	79.5–96.2
Vietnamese (2)	4.1	4.5	4.4	4.3	31/40	77.5%	61.6–89.2
Korean (2)	3.8	4.2	4.2	4.4	33/40	82.5%	67.2–92.7
Farsi (2)	3.1	3.7	3.6	3.7	27/40	67.5%	50.9–81.4
ALL	4.2	4.4	4.3	4.3	330/400	82.5%	78.4–86.1

**Table 3:** Selected Examples of Gross Errors in Translation

English statement	Translation
“You can take over the counter ibuprofen as needed for pain.”	Armenian: “You may take anti-tank missile as much as you need for pain.”
“Your Coumadin level was too high today. Do not take any more Coumadin until your doctor reviews the results.”	Chinese: “Your soybean level was too high today. Do not take anymore soybean until your doctor reviews the results.”
“Do not blow your nose or put pressure on your facial fracture.”	Chinese: The character chosen for blow is more commonly used in relation to “the wind blowing”
	Farsi: “Do not explode your nose because it could put pressure on the break in your face.”

## DISCUSSION

As the practice of using GT for medical communication becomes more widespread, it is crucial that we understand its accuracy and limitations in the medical setting. Khoong et al. studied the use of GT for ED discharge instructions in Spanish and Chinese. They had professional translators rate the translations for accuracy and potential harm. They reported 8% inaccuracies in Spanish and 19% in Chinese translations and potential harm in 2% of Spanish discharge instructions and 8% of Chinese. The authors concluded that GT had high accuracy and GT translations can supplement but not replace written English instructions and should include a warning about potentially inaccurate instructions.^10^ Our accuracy rates for these two languages as assessed by volunteers from the community were almost identical (Spanish 6% inaccuracies and Chinese 18%) to those of professional translators. This is important information for future work in this area as the difference between patient perception of machine translations and a professional translator’s perception has been an ongoing question. While we, like Khoong et al., found the overall accuracy of GT to be better than historically reported, this did not hold true for all languages. Alarmingly, Armenian and Farsi, which are commonly spoken in our community, had accuracy rates of 55 and 67.5% respectively.

Beyond the variability in the accuracy rates, we also found several issues related to GT use that may not be appreciated by clinicians with limited knowledge of the target languages. For instance, when we first created our GT worksheets in Farsi, we found that the directionality of the written language was not accounted for by the software, i.e., that Farsi is written right to left. When we presented the Farsi GT worksheet to the initial volunteer, it was transposed to left to right by GT and was illegible. If these were real discharge instructions, they would be unreadable to the patient. Furthermore, volunteers mentioned potential issues with traditional versus modern Chinese writing systems and Persian versus Afghan versus Tajiki Farsi. It is easy to imagine a well-meaning provider Google Translate-ing instructions into one of these languages without awareness of these potential issues and potentially causing harm.

The important implication of our study is that, despite recent reports of improvement in accuracy and the suggestion that GT has a role for use in the clinical setting, we found that GT accuracy varies substantially by language and is not yet a reliable tool in the clinical setting. Even for languages in which the accuracy is high, there is still the potential for important inaccuracies and the potential for patient harm. The best practice remains to use prewritten, professionally translated discharge instructions in the patient’s native language for general information about a diagnosis when such handouts are available in the electronic health record. For patient-specific instructions, clinicians should hand the patient a copy of their discharge instructions in English and use an interpreter to have the instructions verbally interpreted to the patient. While the interpreter is on the line, use a teach-back to be sure the patient understands the information.

## LIMITATIONS

Our study is limited in that it may overestimate accuracy rates as the participants had lived in the USA for long periods of time and had high levels of acculturation and may not be representative of the understanding of recent immigrants who have the added barrier of lack of familiarity with our health system. All of our volunteers were literate in both English and the target language and we did not formally assess health literacy. This may also cause an overestimation of the accuracy levels that would be reported by participants with limited literacy. Similarly, we used bilingual participants whose language abilities may not accurately represent the understanding of patients who are monolingual in a language other than English. GT also uses an artificial intelligence algorithm that is always changing. It is possible that further improvements have been made since the time of this study.

## CONCLUSIONS

Accuracy rates of translations by GT for ED discharge instructions vary by language. Although the future of written translation in hospitals is likely machine translation, GT is not ready for prime time use in the emergency department.
